# Coral transplantation triggers shift in microbiome and promotion of coral disease associated potential pathogens

**DOI:** 10.1038/srep11903

**Published:** 2015-07-06

**Authors:** Jordan M. Casey, Sean R. Connolly, Tracy D. Ainsworth

**Affiliations:** 1 Australian Research Council Centre of Excellence for Coral Reef Studies, Townsville, QLD, Australia; 2 College of Marine and Environmental Sciences, James Cook University, Townsville, QLD, Australia

## Abstract

By cultivating turf algae and aggressively defending their territories, territorial damselfishes in the genus *Stegastes* play a major role in shaping coral-algal dynamics on coral reefs. The epilithic algal matrix (EAM) inside *Stegastes*’ territories is known to harbor high abundances of potential coral disease pathogens. To determine the impact of territorial grazers on coral microbial assemblages, we established a coral transplant inside and outside of *Stegastes*’ territories. Over the course of one year, the percent mortality of transplanted corals was monitored and coral samples were collected for microbial analysis. As compared to outside damselfish territories, *Stegastes* were associated with a higher rate of mortality of transplanted corals. However, 16S rDNA sequencing revealed that territorial grazers do not differentially impact the microbial assemblage of corals exposed to the EAM. Regardless of *Stegastes* presence or absence, coral transplantation resulted in a shift in the coral-associated microbial community and an increase in coral disease associated potential pathogens. Further, transplanted corals that suffer low to high mortality undergo a microbial transition from a microbiome similar to that of healthy corals to that resembling the EAM. These findings demonstrate that coral transplantation significantly impacts coral microbial communities, and transplantation may increase susceptibility to coral disease.

Microbial diversity is essential for the functioning and resilience of terrestrial and marine ecosystems^1^. On coral reefs, microbial communities influence biogeochemical and ecological processes such as nutrient cycling and larval recruitment^2^,^3^. Reef-building corals are host to well-studied obligate symbionts in the genus *Symbiodinium*, but less is known about the diverse assemblages of bacteria that associate with corals^4^. Microbial communities are hypothesized to confer many benefits to their coral hosts, such as energy provision, photosynthesis, nitrogen fixation, and the prevention of infection. However, under disturbances and stressful environmental conditions, shifts in the coral microbiome have been linked to the degradation of coral reefs^5^. Yet, current research does not adequately address the potential impacts of trophic interactions on microbial communities on coral reefs.

In the marine environment, benthic microbial communities are influenced by an array of abiotic and biotic factors, including nutrient fluxes and benthic-feeding organisms^6^,^7^. Territorial damselfishes are abundant and important engineers of the reef benthos and play a key role in benthic dynamics^8^,^9^,^10^. These fishes exhibit several key behaviors that extensively modify benthic structure in their territories: grazing turf algae, weeding unpalatable macroalgae, coral-pecking to further propagate turf algae, and engaging in constant aggression to protect their algal resources from intruders^11^,^12^,^13^. Although turf algae is omnipresent across the reef benthos, territorial damselfishes cultivate a notably thicker turf inside their territories, and damselfishes in the genus *Stegastes* maintain low-diversity algal turfs on the benthos and coral skeletons^9^,^14^. Thus, damselfish territories are dominated by the epilithic algal matrix (EAM), a conglomeration of turf algae, juvenile macroalgae, detritus, invertebrates, and bacterial assemblages^15^,^16^,^17^,^18^.

The EAM is known to have negative effects on coral growth and survival both inside and outside of damselfish territories^19^,^20^,^21^,^22^,^23^,^24^; therefore, due to their cultivation of a thick EAM, the impact of territorial grazers on corals is largely negative. Territorial grazers have been shown to inhibit coral recruitment^25^, negatively impact the abundance of juvenile corals^26^, and cause mortality in adult corals^11^. The EAM inside *Stegastes*’ territories has also been shown to harbour potential pathogens linked to black band disease (BBD)^7^. Due to the elevated levels of potential coral disease pathogens in the EAM, territorial grazers may indirectly promote disease and mortality in corals inside their territories. Direct contact-mediated coral-algal interactions may cause toxicity or hypoxia in coral tissues, thus facilitating the invasion of opportunistic pathogens. This stress-induced increase in potential pathogens may ultimately lead to coral disease or death^18^.

Although corals and algae have a highly antagonistic relationship on coral reefs, they are known to harbour distinct microbial communities^20^, and corals can outcompete turf algae in areas of low anthropogenic disturbance^27^. While territorial damselfishes in the genus *Stegastes* have been shown to negatively impact corals, the relationship between damselfishes and corals is variable depending on region and habitat, and damselfishes have also been reported to increase coral recruitment^28^ and facilitate adult coral survival^29^,^30^,^31^,^32^,^33^. Therefore, the high occurrence of coral disease-associated bacteria in the EAM inside *Stegastes*’ territories is not necessarily indicative of the composition of neighboring coral microbial assemblages. Thus, this study aims to determine the coral survivorship and the microbial composition of corals within territories of damselfishes in the genus *Stegastes.* Specifically, we determined the impact of *Stegastes* on the survival of transplanted corals and analyzed how coral transplantation inside and outside of *Stegastes*’ territories affects coral microbial communities.

## Methods

### Study site and species

This study took place at Lizard Island in the northern GBR, Australia (14°41′5”S, 145°26′55”E) from February 2012 to August 2013. The study site was on the back reef in the lagoon between Palfrey and South Island at a depth of 1–3 m.

*Stegastes nigricans* and *Stegastes apicalis* (f. Pomacentridae), two intensive territorial grazers, were our damselfish study species. *S. apicalis* occurs at a depth of 1–15 m and reaches up to 15 cm (total length). *S. nigricans* occurs at the depth of 1–12 m and reaches up to 14 cm (total length). Both species form social groups (termed “colonies”) made up of several contiguous territories, where each territory belongs to an individual adult damselfish.

*Acropora muricata* was our coral study species. It is a common staghorn coral with aborescent branching in shallow reefs around Lizard Island, especially lagoon and back reef habitats. *Stegastes* typically form colonies within or around outcrops of *A. muricata.*

### Coral baseline samples

To determine the impact of territorial damselfishes on coral microbial communities, we first collected coral branches from outside and inside damselfish territories. In the field, ten 15 cm coral branches were collected from control plots outside damselfish territories, ten from inside *S. apicalis*’ territories, and ten from inside *S. nigricans*’ territories. For the collections from *S. apicalis*’ and *S. nigricans*’ territories, branches were taken across two damselfish colonies, and each branch came from a different damselfish territory.

### Coral transplant

To determine the effects of EAM exposure and damselfish farming behaviours on coral fragments over time, we set up a coral transplant outside and inside damselfish territories. Due to the higher success rate of coral transplantation of *A. muricata* when medium to large-sized fragments (10–20 cm) are used^34^, we sourced 120 15-cm fragments of *A. muricata* from outside damselfish territories. To minimize handling time, coral fragments were briefly transported to large seawater bins for labeling at the field site, then immediately transplanted using marine epoxy. Coral fragments were directly transplanted rather than allocating a recovery period in holding tanks because placing corals in holding tanks after fragmentation can substantially alter bacterial assemblages^35^ and experimental injuries (such as wounding from fragmentation) have been shown to have a limited impact on the coral immune response^36^. Forty fragments were transplanted in control plots outside of damselfish territories, forty fragments were transplanted inside *S. apicalis*’ territories, and forty fragments were transplanted in *S. nigricans*’ territories. Coral fragments were distributed randomly across treatments with respect to the source colonies. Two transplanted corals were placed in each *Stegastes*’ territory, and the transplanted corals were distributed across two *S. apicalis*’ colonies and two *S. nigricans*’ colonies, which were the same colonies that were used for baseline sample collections.

After six months, the percent mortality (percentage of tissue loss) of each coral fragment was estimated, and ten coral fragments were sampled from each treatment for microbial analyses. Percent mortality estimates and microbial sampling (ten coral fragments from each treatment) were repeated after one year. Thus, we sampled a total of 90 coral fragments: 30 baseline samples, 30 samples after six months of transplantation, and 30 samples after one year of transplantation. Of each 30 samples, ten were from control plots outside of damselfish territories, ten were from *S. apicalis*’ territories, and ten were from *S. nigricans*’ territories.

### Microbial processing

After collection, coral fragments were immediately snap-frozen in liquid nitrogen, stored at −80°C, and transported to James Cook University for processing and DNA extractions. Samples were homogenized under liquid nitrogen. When tissue fragments suffered partial to high mortality, care was taken to homogenize only live tissue sections of the coral fragments (dead tissue and algae coverage was excluded). See Casey *et al.*^7^ for methods of DNA isolation, quality control, PCR, and 16S rDNA sequencing.

### Coral mortality data analysis

**Figure 1:**
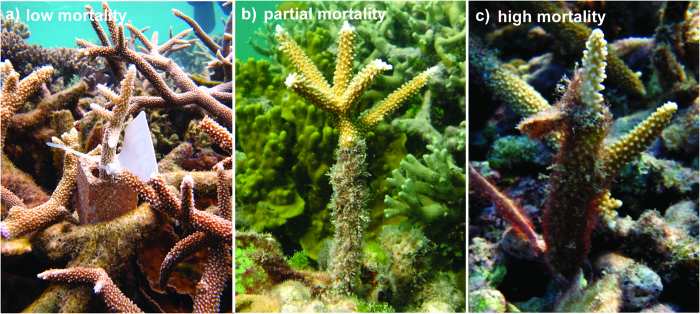
Mortality of transplanted corals in *Stegastes*’ territories after six months. (**a**) Low mortality (0–20%), (**b**) partial mortality (20–80%), and (**c**) high mortality (80–99%). Photographs taken by J.M.C.

To analyze the effect of intensive territorial grazers on the mortality of transplanted corals, we used Akaike’s Information Criterion (AIC) to compare the fit of three generalized linear models (GLM) to our data (Table 1). Due to preferential removal of coral fragments with low mortality for microbial analysis after six months of transplantation, we focused on mortality only over the first six month period to avoid biases associated with sample removal. For each model, the response was multinomial (low mortality, partial mortality, and high mortality in transplanted coral): low mortality was defined as 0–20% loss of tissue from the coral branch, partial mortality was defined as 20–80% loss of tissue from the coral branch, and high mortality was defined as 80–99% loss of tissue from the coral branch (Fig. 1). In the first GLM, the fixed effect was the presence or absence of a damselfish territory, separating the effects of damselfish species (control plots with no damselfish territory *versus S. apicalis*’ territories and *S. nigricans*’ territories). In the second GLM, the fixed effect was also the presence or absence of a damselfish territory, without separating the effects of damselfish species (control plots with no damselfish territory *versus* damselfish territories). The third GLM was an intercept-only model with no treatment effects. The analysis was conducted with the package *nnet*^37^ using the software program R.

### Microbial data analysis

The sequence data were processed using a proprietary analysis pipeline at MR DNA and re-analyzed with Quantitative Insights Into Microbial Ecology (QIIME)^38^. For both the proprietary analysis pipeline at MR DNA and QIIME, sequences were depleted of barcodes and primers, and short sequences (<200 bp) and sequences with homopolymer runs exceeding 6 bp were removed. The average read length was 437 bp after primer and barcode removal. See Casey *et al.*^7^ for further methods of denoising, normalization, definition of operational taxonomic units (OTUs), and taxonomical assignments with QIIME.

We assessed the beta-diversity of the coral and EAM microbial communities (EAM samples were collected concurrently at the same study site; data published by Casey *et al.*^7^) inside and outside of damselfish territories with QIIME using a weighted UniFrac analysis. A principal coordinates analysis (PCoA) was generated from the UniFrac distances^39^. A PCoA was generated from weighted UniFrac distances and plotted in two dimensions.

Individual OTUs were assigned into three categories: autotrophs, heterotrophs and potential pathogens, based on literature reviews (Supplementary Table S1). All genera with a less than two percent relative abundance were excluded from the analyses (Supplementary Tables S2-S10). Our data included twenty-one genera that are considered potential pathogens for a broad array of hosts; however, among these genera, four are specifically linked to coral disease. These four coral-specific potential pathogens made up 26.4% percent relative abundance of the potential pathogen category, and we focused on these coral disease associated potential pathogens in a further analysis.

We fit GLMs to analyze the differences in the relative abundances of autotrophs, heterotrophs, potential pathogens, and potential coral disease pathogens in coral microbial communities outside of damselfish territories as compared to inside *S. apicalis’* territories and *S. nigricans*’ territories and across the three sampling periods (baseline, six month of transplantation, and one year of transplantation). We used a quasi-binomial error structure to account for the fact that the response variable (relative abundance) varied continuously between zero and one. The fixed effects included treatment (control plots outside of damselfish territories, inside *S. apicalis’* territories, and inside *S. nigricans*’ territories) and time (baseline, transplantation after six months, and transplantation after one year) and their interactions. Thus, these models simultaneously analyzed the effects of damselfish presence as well as time of transplantation on the metabolic groupings of coral bacterial assemblages. Since we employed a quasi-binomial error structure in this analysis, likelihood-based model selection, such as AIC, could not be used. Instead, we employed a quasi-likelihood procedure based on adjusted model deviances, which utilizes the standard *F* distribution as the null distribution (Table 2, see Zuur *et al.*^40^). The analysis was conducted with the packages *lme4*^41^ and *arm* using the software program R.

## Results

### Mortality of transplanted corals

**Figure 2:**
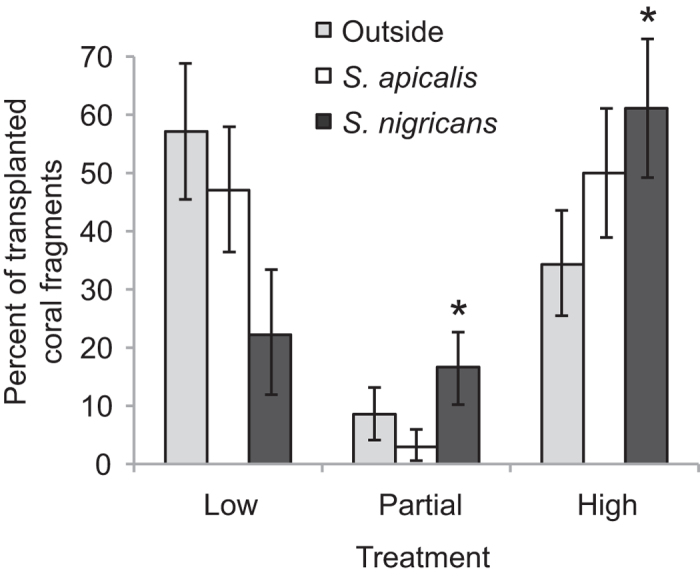
Percent of transplanted coral fragments (±SE) that suffered mortality after six months of transplantation. Mortality is categorized as low mortality (0–20%), partial mortality (20–80%), and high mortality (80–99%). Treatments include control plots outside damselfish territories (35 fragments), inside *S. apicalis’* territories (34 fragments), and inside *S. nigricans’* territories (36 fragments). Bars represent means and standard errors of percentages of fragments in each mortality category across control plots outside damselfish territories and inside different damselfish territories (*n* = 20 territories in each case). Asterisks indicate significant values (p < 0.05).

Model selection indicated that the best model of coral mortality included the effects of both damselfish species by comparing control plots with no damselfish territory to *S. apicalis*’ territories and *S. nigricans*’ territories. Specifically, after six months, corals transplanted inside damselfish territories suffered a higher mortality (loss of tissue) than corals outside damselfish territories, with coral fragments inside *S. nigricans*’ territories exhibiting a stronger estimated response than fragments inside *S. apicalis*’ territories (Table 1, Fig. 1, and Fig. 2).

### Coral microbial communities

**Figure 3:**
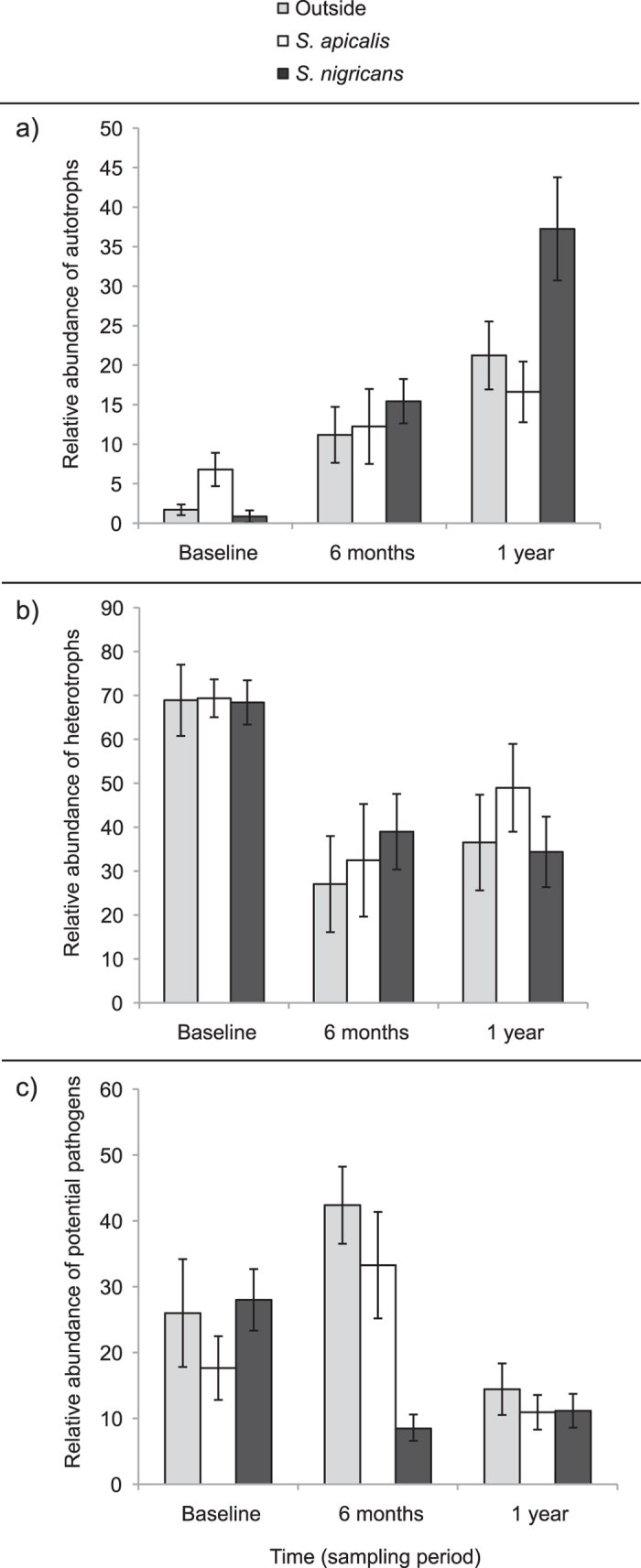
Relative abundances of autotrophs, heterotrophs, and potential pathogens in coral fragments. The relative abundance (±SE) of (**a**) autotrophs, (**b**) heterotrophs, and (**c**) potential pathogens according to damselfish presence (control plots outside damselfish territories, inside *S. apicalis’* territories, and inside *S. nigricans’* territories) and time after transplantation (baseline coral fragments, transplanted coral fragments after six months, and transplanted coral fragments after one year).

Bacterial 16S rDNA gene amplicon sequencing retrieved 235,388 high-quality sequence reads from 77 coral samples (DNA extractions and sequencing were successful for 77 coral samples out of the 90 collected coral fragments). Sequence reads were normalized to 960 reads per sample to allow for comparison between samples and bacterial community patterns, which further excluded two samples, resulting in 75 coral samples. In an analysis of all bacterial phylotypes, genera were assigned into the metabolic groupings of autotrophs, heterotrophs, and potential pathogens (Supplementary Table S1), revealing that microbial communities in the coral fragments shift according to the presence of damselfish territories and over the course of one year of transplantation (Fig. 3). Model selection indicated that the best model for the relative abundance of autotrophs included the main effects of treatment (damselfish effects) and time, but no interaction (Table 2). As compared to baseline coral microbial communities, there were significant increases in the relative abundance of autotrophs after six months (p = 0.001) and one year (p = 0.008) of transplantation (Fig. 3). Further, as compared to corals transplanted outside of damselfish territories, corals inside *S. nigricans*’ territories had significantly lower relative abundances of autotrophs (p = 0008). For heterotrophs, model selection indicated that the best model included the effect of time only (Table 2). As compared to baseline coral microbial communities, there were significant decreases in the relative abundance of heterotrophs after six months (p < 0.001) and one year (p < 0.001) of transplantation (Fig. 3). Lastly, model selection indicated that the best model for potential pathogens included the full model: effects of treatment (damselfish effects), time, and their interactions (Table 2). The relative abundance of potential pathogens was impacted by a significant interaction (p = 0.002). Visual inspection suggests that changes in potential pathogens across treatments and time were driven by larger increases over the first six months in control fragments relative to those in damselfish territories, particularly those of *S. nigricans* (Fig. 3).

### Potential coral disease pathogens

**Figure 4:**
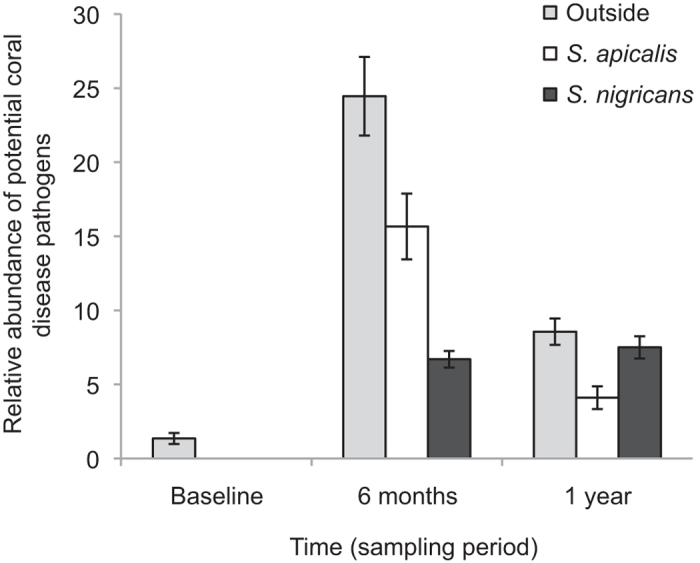
Relative abundances of potential coral disease pathogens in coral fragments. The relative abundance (±SE) of coral disease associated potential pathogens according to damselfish presence (control plots outside damselfish territories, inside *S. apicalis’* territories, and inside *S. nigricans’* territories) and time after transplantation (baseline coral fragments, transplanted coral fragments after six months, and transplanted coral fragments after one year).

Model selection indicated that the best model for the relative abundance of potential coral disease pathogens included the main effects of treatment (damselfish effects) and time, but no interaction (Table 2). As compared to baseline coral fragments, acroporid fragments experienced significantly higher relative abundances in potential pathogens associated with coral disease after six months (p = 0.001), and to a lesser extent, after one year (p = 0.008) of transplantation (Fig. 4). The potential coral disease pathogens were in the genera *Geitlerinema*, *Leptolyngbya*, *Oscillatoria,* and *Sphingomonas*^42^,^43^. Of these coral-disease associated potential pathogens, *Geitlerinema*, *Leptolyngbya*, and *Oscillatoria* are associated with BBD^42^. Also, as compared to no-damselfish controls, there were significantly lower relative abundances of potential pathogens associated with coral disease in coral fragments inside *S. nigricans*’ territories (p = 0.008), but the lower abundances inside *S. apicalis*’ territories did not differ significantly from controls (p = 0.073, Fig. 4).

## Discussion

This study reveals that the presence of territorial damselfishes increases the rate of mortality of corals relocated to within territories. As compared to benthic plots outside damselfish territories, *S. nigricans* trigger the highest rate of mortality in transplanted corals, with *S. apicalis* causing an intermediate rate of mortality between plots outside of damselfish territories and *S. nigricans*’ territories. It has been shown that *S. nigricans* engage in more intensive farming behaviours than *S. apicalis*^7^; therefore, it is likely that *S. nigricans*’ intensive cultivation of the EAM or direct polyp mortality by coral-pecking may outcompete stressed, transplanted corals. Surprisingly, despite these higher rates of mortality, *Stegastes* do not have a differential impact on coral microbial communities. While previous work has shown that there are higher relative abundances of coral disease pathogens in the EAM inside *Stegastes’* territories^7^, this study demonstrates that shifts in bacterial assemblages in corals after transplantation are not directly related to the impact of territorial damselfishes.

**Figure 5:**
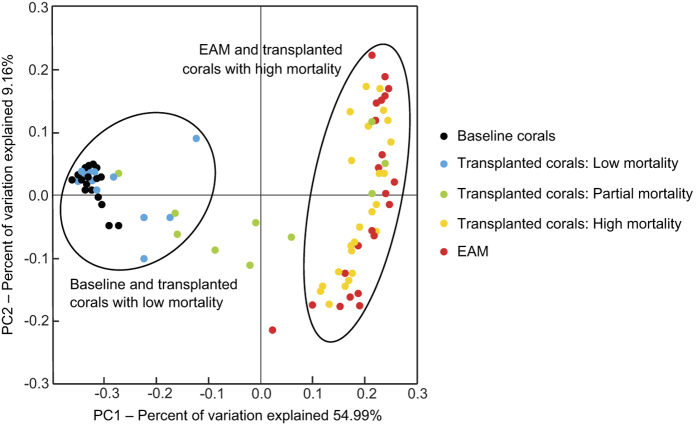
Principal coordinates analysis (PCoA) showing the percent of variation explained in the microbial community. Treatments include baseline coral fragments, transplanted corals with low mortality (0–20%), transplanted corals with partial mortality (20–80%), transplanted corals with high mortality (80–99%), and EAM samples (data published in Casey *et al.*^7^) outside damselfish territories, inside *S. apicalis*’ territories, and inside *S. nigricans*’ territories. The ellipses represent distinct clustering of the baseline corals, transplanted corals, and EAM samples.

However, the damage caused by coral transplantation is found to impact the microbial community of corals. Six months after coral transplantation, over fifty percent of the coral fragments suffered partial to high mortality. As a result of this high rate of mortality, coral transplantation both inside and outside damselfish territories also triggered a shift in the coral microbiome that is evident up to a year after the initial transplantation. To further analyze how the microbiome of transplanted corals shift over time as a function of percent coral mortality, we examined the similarity of transplanted coral microbial communities to EAM microbial communities (EAM data published by Casey *et al.*^7^). This analysis reveals a transition in the microbial community of baseline coral samples and the healthy tissue of transplanted corals with low (0–20%) mortality to transplanted corals with partial (20–80%) mortality to transplanted corals with high (80–99%) mortality (Fig. 5). The microbial community of transplanted corals with high mortality is more similar to the EAM microbial community than to the baseline coral microbial community. Exposure to the benthos, and consequently the EAM, has a transformative effect on microbial communities of transplanted corals that suffer partial to high mortality, as the coral tissue undergoes a microbial transition from an association with healthy corals to an association with the EAM. This microbial shift, in which coral microbial communities increasingly resemble the EAM microbial community, has major implications for benthic microbial assemblages. When benthic microbial communities become homogenous, from the EAM to corals, this loss of microbial diversity may lead to decreased resilience against potential coral disease pathogens^44^,^45^,^46^.

Previous work shows that there are higher relative abundances of BBD pathogens in the EAM inside *Stegastes’* territories as well as a higher occurrence of BBD inside *S. nigricans*’ territories^7^. This study reveals that the higher relative abundance of potential coral disease pathogens in the EAM inside *Stegastes*’ territories may opportunistically cause BBD in acroporids, but it does not demonstrate nor predict shifts in the overall coral microbial assemblage in *A. muricata* inside *Stegastes*’ territories. The fact that BBD pathogens are omnipresent, albeit in lower abundances, across the reef benthos allows them to opportunistically colonize stressed transplanted corals, regardless of *Stegastes* presence or absence. It is known that even healthy corals have potential pathogens in their bacterial assemblages, and under changing environmental conditions, a commensal may transition to a pathogenic state^44^. Here, we find that one prevalent taxon among our samples has been previously linked to disease in corals. Bacteria in the genus *Ruegeria* were consistently present within 50 percent of all coral fragments and previously have been associated with both healthy corals and coral lesions resulting from Yellow Band Disease^46^. *Ruegeria* also undergoes horizontal gene transfer, which may help hosts and microbial associates adapt to environmental challenges in short time periods^47^,^48^. Despite the suggestion of a possible link between *Ruegeria* and the promotion of coral disease^46^,^49^,^50^, the common occurrence of *Ruegeria* in baseline and transplanted corals suggests that the role of these bacteria is a commensal in the current study.

A considerable number of studies have investigated the efficacy of coral transplantation as a means for coral reef restoration by examining how transplantation affects coral growth, mortality and physiology^51^,^52^,^53^,^54^,^55^,^56^,^57^. While it is known that environmental stressors^5^,^58^,^59^,^60^,^61^ and coral disease^49^,^50^,^62^,^63^,^64^,^65^,^66^ cause changes in coral microbial communities, no previous study has analyzed the impact of coral transplantation on coral microbial communities. Due to the use of coral transplantation for coral reef restoration^53^,^54^,^55^,^56^,^57^,^67^,^68^,^69^,^70^,^71^,^72^, this paper demonstrates how transplantation may negatively impact the survival and health of corals. Since microbes are key players in coral health, it is imperative to consider microbial communities when examining the utility of conservation measures such as coral transplantation^1^.

Here, we show that coral fragments undergo higher rates of mortality inside damselfish territories, but territorial grazers do not differentially affect the microbial communities of transplanted corals. Rather, the damage caused by coral transplantation leads to a shift in the microbial community toward an increase in potential coral disease pathogens, especially those linked to BBD^42^, which is independent of territorial grazer presence or absence. The increase in potential pathogens in transplanted corals suggests that transplanted corals may be more susceptible to coral disease under certain stressful environmental conditions, such as an increase in sea surface temperature or nutrient fluxes. This study highlights the importance of examining ecological interactions beyond trends of macro-organisms and demonstrates how microbial communities provide essential information about coral health and resilience^1^,^4^,^73^.

## Additional Information

**Accession codes:** The DNA sequence data appears in the National Center for Biotechnology Information (NCBI) under BioProject submission ID SUB924371. 

**How to cite this article**: Casey, J. M. *et al.* Coral transplantation triggers shift in microbiome and promotion of coral disease associated potential pathogens. *Sci. Rep.* **5**, 11903; doi: 10.1038/srep11903 (2015).

## Supplementary Material

Supplementary Information

## Figures and Tables

**Table 1:** Model selection among three generalized linear models with a multinomial distribution showing the effects of territorial damselfishes on the partial mortality (20–80%) and high mortality (80–99%) of transplanted corals, including a comparison of a) control plots without damselfish present (Control), *S. apicalis*’ territories, and *S. nigricans*’ territories, b) control plots without damselfish present and damselfish territories (Damselfish), and c) an intercept-only model with no treatment effects.

**Model**	**Mortality**	**Variable**	**Coefficients**	**Std. Error**	**p-value**	**AIC**
a) Damselfish species	Partial	Control	−1.897	0.619		181.74
		*S. apicalis*	−0.875	1.202	0.234	
		*S. nigricans*	1.609	0.822	**0.027**	
	High	Control	−0.798	0.401		
		*S. apicalis*	0.798	0.535	0.070	
		*S. nigricans*	1.492	0.590	**0.007**	
b) Damselfish grouped	Partial	Control	−1.897	0.619		184.27
		Damselfish	0.665	0.754	0.190	
	High	Control	−0.798	0.401		
		Damselfish	1.086	0.484	**0.014**	
c) Intercept only	Partial	Intercept	−1.481	0.350	**<0.001**	185.66
	High	Intercept	−0.071	0.217	**<0.001**	

P-values are for individual effects and represent tests of the null hypothesis that the relevant treatment differs from the control. Significant values (p < 0.05) are displayed in bold.

**Table 2:** Model selection for four generalized linear models with quasi-binomial error structures for the relative abundances of autotrophs, heterotrophs, potential pathogens, and potential coral disease pathogens in coral fragments in control plots outside *Stegastes*’ territories as compared to coral fragments inside *S. apicalis’* territories and *S. nigricans’*territories (damselfish effects).

**Energetic grouping**	**Model selection**	**Difference in deviances**	**Degrees of freedom**	**F-value**	**p-value**
Autotrophs	Full model vs. Main effects only	0.630	4	1.460	0.224
	Main effects vs. Damselfish effects only	7.952	2	35.960	**<0.001**
	Main effects vs. Time effects only	1.312	2	5.935	**0.004**
Heterotrophs	Full model vs. Main effects only	0.389	4	0.272	0.895
	Main effects vs. Damselfish effects only	8.685	2	12.692	**<0.001**
	Main effects vs. Time effects only	0.354	2	0.517	0.598
Potential pathogens	Full model vs. Main effects only	1.487	4	2.958	**0.026**
	Main effects vs. Damselfish effects only	1.256	2	4.502	**0.014**
	Main effects vs. Time effects only	1.228	2	4.402	**0.016**
Potential coral disease pathogens	Full model vs. Main effects only	0.629	4	1.535	0.202
	Main effects vs. Damselfish effects only	4.661	2	22.103	**<0.001**
	Main effects vs. Time effects only	0.897	2	4.255	**0.018**

Sampling periods include baseline coral fragments as compared to transplanted corals after six months and transplanted corals after one year (time effects). The full models include the damselfish effects, time effects, and their interactions. Model selection was performed with a quasi-likelihood procedure based on adjusted model deviances, which utilizes the standard *F* distribution. P-values test the null hypothesis that the simpler model of the two being compared is true; thus, bolded values (p < 0.05) indicate rejection of the simpler model in favor of the more complex one.
