# Supplementary material for: Coral transplantation triggers shift in microbiome and promotion of coral disease associated potential pathogens
Source: Sci Rep. 2015 Jul 6;5:11903. doi: 10.1038/srep11903 (PMC4491727; doi:10.1038/srep11903)
Supplement: Supplementary Information [file srep11903-s1.pdf]

Coral transplantation triggers shift in microbiome and promotion of coral disease associated potential pathogens

Jordan M. Casey*, Sean R. Connolly, Tracy D. Ainsworth

Supplementary Table S1 | Assignments of bacterial genera into metabolic categories (autotrophs, heterotrophs, and potential pathogens). "R" denotes reference for metabolic grouping.

Autotrophs	R	Heterotrophs	R	Potential pathogens	R
Anabaena	S1	*Afifella*	S25	*Acinetobacter*	S65
Arthrospira	S2	*Alicyclobacillus*	S26	*Aeromonas*	S66
Calothrix	S3	*Anderseniella*	S27	*Balneatrix*	S67
Chamaesiphon	S4	*Candidatus* Microthrix	S28	*Bartonella*	S68
Chrococcidiopsis	S5	*Comamonadaceae*	S29	*Burkholderia*	S69
Coleochaete	S6	*Congregibacter*	S30	*Candidatus* Amoebophilus	S70
Crenothrix	S7	*Cystobacterineae*	S31	*Chryseobacterium*	S71
Gloeobacter	S8	*Endozoicomonas*	S32	*Corynebacterium*	S72
Leptospirillum	S9	*Enhydrobacter*	S33	*Escherichia*	S73
Microcrocoleus	S10	*Epulopiscium*	S34	*Geitlerinema**	S74
Nitrosococcus	S11	*Geobacter*	S35	*Gordonia*	S75
Pedinomonas	S12	*Haliea*	S36	*Halomonas*	S76
Pellia	S13	*Kangiella*	S37	*Leptolyngbya**	S74
Pleurocapsa	S14	*Labrenzia*	S38	*Micrococcus*	S77
Prochlorococcus	S15	*Lactobacillus*	S39	*Mycoplasma*	S78
Prochlorothrix	S16, S17	*Leucothrix*	S40	*Oscillatoria**	S74
Pseudanabaena	S18	*Magnetospirillum*	S41	*Propionibacterium*	S79
Rhodovibrio	S19	*Marinobacter*	S42	*Pseudomonas*	S80
Rivularia	S20	*Massilia*	S43	*Ralstonia*	S81
Scherffelia	S21	*Mesorhizobium*	S44	*Sphingomonas**	S82
Spirulina	S22	*Methylobacterium*	S45	*Staphylococcus*	S83
Synechococcus	S23	*Microbulbifer*	S38		
Trichodesmium	S24	*Muricauda*	S46		
		Nannocystineae	S31		
		Neptunomonas	S47		
		Nitratireductor	S48		
		Oceanospirillum	S49		
		Pelagibius	S50		
		Pelobacter	S51		
		Phyllobacteriaceae	S52		
		Pseudovibrio	S38		
		Rheinheimera	S53		
		Rhodobacteraceae	S54		
		Rhodobium	S55		
		Rhodopirellula	S56		
		Rhodospirillaceae	S57		
		Rhodovulum	S58		
		Rubrobacter	S59		
		Ruegeria	S60		
		Salinicoccus	S61		
		Thalassospira	S62		
		Wenxinia	S63		
		Wolbachia	S64		

*****Geitlerinema*, *Leptolyngbya*, *Oscillatoria,* and *Sphingomonas* are coral-specific potential pathogens. The first three genera are associated with black band disease.

Supplementary Table S2 | Assignments of OTUs with at least a two percent relative abundance to genus or next lowest taxonomical unit and their respective relative abundance in each baseline coral sample from control plots.

**Taxa**	**Coral Sample**							
	1	2	3	4	5	6	7	8
Enhydrobacter	7.06	0.00	0.00	0.00	0.00	0.00	0.00	0.00
Pseudomonas	27.82	1.57	2.97	0.11	0.39	0.79	0.13	0.00
Candidatus Amoebophilus	0.00	0.00	0.00	0.00	4.69	16.79	0.13	53.63
Microbulbifer	0.00	0.23	0.12	4.63	0.00	0.73	0.39	0.00
Neptunomonas	2.82	49.71	54.44	5.99	35.09	36.62	19.34	1.34
Gloeotrichia	0.00	0.00	0.00	0.00	4.20	2.49	2.50	4.39
Marinobacter	0.42	16.29	13.54	0.32	6.74	5.54	3.68	0.76
Staphylococcus	3.25	0.02	0.02	0.00	0.00	1.09	0.79	0.00
Rubrobacter	0.00	0.00	0.00	0.00	2.15	0.00	0.00	0.00
Sphingomonas	0.00	0.00	0.00	0.00	0.00	0.00	0.00	10.50
Propionibacterium	5.79	0.00	0.02	0.00	3.13	0.00	0.26	1.72
Geitlerinema	0.00	0.17	0.21	0.21	0.29	0.30	1.05	8.59
Congregibacter	0.00	0.90	0.06	13.04	0.00	2.68	0.00	0.00
Methylobacterium	18.36	0.00	0.00	0.11	9.87	0.00	0.39	0.00
Pelobacter	0.00	0.00	0.08	0.00	2.93	0.00	0.00	0.00
Corynebacterium	5.65	0.00	0.00	0.00	0.00	0.00	0.00	0.00
Oceanospirillum	0.56	8.68	13.36	69.40	8.80	23.60	23.55	1.91
Lactobacillus	0.00	0.00	0.00	0.00	0.00	0.00	2.76	0.00
Rheinheimera	24.72	0.00	0.00	0.00	0.00	0.00	0.00	0.00
Halomonas	0.56	21.27	13.21	1.05	8.99	5.29	3.42	0.00
Alicyclobacillus	2.54	0.13	0.02	0.95	8.70	0.61	26.58	14.69
Thalassospira	0.00	0.00	0.00	0.00	0.00	0.00	3.29	0.00
Aeromonas	0.00	0.00	0.00	0.00	0.00	0.00	2.11	0.00

Supplementary Table S3 | Assignments of OTUs with at least a two percent relative abundance to genus or next lowest taxonomical unit and their respective relative abundance in each baseline coral sample from *S. apicalis*' territories.

Taxa	Coral Sample						
	1	2	3	4	5	6	7
Rhodovibrio	0.00	0.00	0.00	0.00	3.28	0.00	0.00
Pseudomonas	0.40	10.64	0.73	1.48	1.50	2.19	1.61
Candidatus Amoebophilus	0.10	0.00	3.85	0.00	5.87	0.00	4.44
Neptunomonas	33.30	2.23	41.28	13.05	12.57	28.69	48.06
Gloeotrichia	9.43	8.42	1.47	0.25	6.01	0.00	1.14
Chryseobacterium	0.00	2.48	0.00	0.00	0.00	0.00	0.00
Marinobacter	1.50	0.00	1.28	6.16	3.83	6.57	11.22
Bartonella	0.00	0.25	0.00	20.69	0.41	0.00	0.00
Gordonia	0.00	7.43	0.00	0.00	0.00	0.00	0.00
Propionibacterium	0.50	0.00	4.40	8.37	0.00	2.99	0.38
Ruegeria	0.00	0.00	0.00	0.00	0.00	2.99	0.00
Prochlorococcus	6.22	0.00	5.69	3.45	2.05	0.20	0.00
Pelobacter	0.00	0.00	0.00	3.69	0.41	1.99	0.03
Corynebacterium	0.00	9.65	0.00	0.00	0.00	0.00	0.06
Salinicoccus	0.00	2.48	0.00	0.00	0.00	0.00	0.00
Oceanospirillum	36.51	25.74	15.96	18.47	55.60	38.25	10.87
Epulopiscium	0.00	0.00	2.20	0.00	0.00	0.00	0.00
Halomonas	1.40	0.00	0.55	5.91	2.05	4.78	18.36
Alicyclobacillus	1.60	0.00	18.72	8.87	2.32	1.39	1.17
Leucothrix	0.00	26.49	0.00	0.00	0.00	0.00	0.00

Supplementary Table S4 | Assignments of OTUs with at least a two percent relative abundance to genus or next lowest taxonomical unit and their respective relative abundance in each baseline coral sample from *S. nigricans*' territories.

**Taxa**	**Coral Sample**								
	1	2	3	4	5	6	7	8	9
Ralstonia	2.31	0.00	0.84	2.02	0.06	0.24	1.28	0.47	3.42
Marinobacter	2.57	17.12	6.90	3.95	15.15	15.26	13.76	13.22	6.12
Pelobacter	0.13	0.19	3.23	3.34	0.14	0.00	0.00	0.00	0.00
Oceanospirillum	8.35	5.07	21.74	12.26	6.56	1.36	2.68	4.75	7.68
Congregibacter	3.98	0.00	0.00	0.00	0.00	0.00	0.00	0.00	0.00
Magnetospirillum	0.00	0.00	0.00	0.00	0.00	0.00	0.00	0.00	23.59
Neptunomonas	20.74	60.37	46.42	40.20	62.99	59.86	54.19	46.58	25.15
Acinetobacter	47.64	8.91	11.63	32.29	7.23	17.64	20.97	21.22	27.27
Candidatus Amoebophilus	9.21	0.00	5.85	0.00	0.00	0.00	0.00	0.00	0.00
Halomonas	0.60	5.74	1.99	1.27	6.67	3.40	5.50	4.43	1.89
Gloeotrichia	0.39	0.00	0.47	0.04	0.00	0.00	0.00	6.88	0.00

Supplementary Table S5 | Assignments of OTUs with at least a two percent relative abundance to genus or next lowest taxonomical unit and their respective relative abundance in each coral sample after six months of transplantation from control plots.

**Taxa**	**Coral Sample**						
	1	2	3	4	5	6	7
Trichodesmium	0.00	0.19	9.99	0.00	1.43	0.00	0.12
Synechococcus	0.34	0.14	3.28	0.00	0.71	0.58	2.55
Marinobacter	12.55	18.07	0.08	0.28	0.89	0.07	0.54
Pelobacter	1.27	2.13	0.00	2.15	0.00	0.00	0.22
Spirulina	0.07	0.00	1.20	0.00	2.32	0.66	0.52
Anabaena	0.48	0.19	2.96	0.00	2.14	12.35	8.49
Geitlerinema	0.11	0.05	0.40	0.00	1.07	2.34	3.15
Oceanospirillum	1.45	2.13	0.00	14.26	0.00	0.00	0.00
Ruegeria	0.41	0.00	2.32	0.00	1.60	0.58	1.00
Afifella	0.04	0.00	0.00	0.00	0.89	4.09	0.86
Neptunomonas	32.48	58.86	0.00	3.18	0.00	0.07	0.02
Acinetobacter	14.00	9.66	0.48	50.59	0.00	0.00	0.06
Oscillatoria	1.90	0.00	27.98	0.00	24.24	37.72	17.63
Bartonella	17.39	0.00	0.08	0.00	0.89	0.07	1.55
Leptolyngbya	0.15	0.07	20.86	0.00	16.40	7.09	10.70
Rhodopirellula	0.00	0.00	0.24	0.14	2.32	1.24	1.22
Mesorhizobium	0.60	0.00	0.16	0.00	2.67	1.17	0.80
Candidatus Amoebophilus	4.54	0.00	0.40	0.21	0.36	0.37	0.30
Pseudanabaena	0.00	0.00	0.00	0.00	5.70	1.17	0.12
Halomonas	3.28	5.53	0.00	0.00	0.18	0.00	0.10
Labrenzia	0.71	0.00	0.56	0.00	1.43	1.39	2.47
Kangiella	0.00	0.00	0.16	9.41	0.00	0.00	0.02
Escherichia	0.00	0.00	0.00	14.67	0.00	0.07	0.00
Rhodovibrio	0.15	0.00	0.56	0.00	0.00	0.66	2.03
Gloeotrichia	2.38	0.00	0.72	0.00	3.92	0.58	4.32
Prochlorothrix	0.07	0.00	1.28	0.00	0.71	0.95	2.17

Supplementary Table S6 | Assignments of OTUs with at least a two percent relative abundance to genus or next lowest taxonomical unit and their respective relative abundance in each coral sample after six months of transplantation from *S. apicalis*' territories.

Taxa	Coral Sample							
	1	2	3	4	5	6	7	8
Trichodesmium	0.00	0.06	0.17	0.00	0.31	2.63	0.30	1.75
Ralstonia	11.02	0.04	0.91	1.78	0.00	0.00	0.00	0.00
Synechococcus	0.25	0.08	0.02	0.00	2.08	3.57	1.69	2.21
Marinobacter	0.33	18.94	21.10	8.08	0.23	0.66	0.70	0.93
Pelobacter	0.00	0.61	0.47	5.37	0.62	0.00	0.30	1.05
Geobacter	3.14	0.00	0.09	0.42	0.00	0.00	0.60	0.00
Gloeobacter	0.00	0.00	0.00	0.00	0.54	0.00	0.20	8.16
Spirulina	0.00	0.00	0.00	0.00	6.01	2.73	2.49	4.55
Anabaena	0.00	0.55	0.00	0.00	8.64	5.93	12.62	8.04
Geitlerinema	0.00	0.00	0.00	0.00	1.93	1.03	2.19	1.63
Oceanospirillum	1.61	3.52	2.70	10.41	0.15	0.00	0.00	0.00
Ruegeria	0.77	0.06	0.00	0.06	2.78	1.41	0.70	1.75
Wolbachia	0.00	0.00	0.00	0.16	2.54	1.41	0.50	0.00
Neptunomonas	0.54	63.77	51.38	39.20	0.00	0.38	0.00	0.12
Acinetobacter	73.11	5.47	14.16	21.36	0.15	0.00	0.10	0.00
Candidatus Microthrix	0.00	0.00	0.00	0.06	2.39	0.56	0.00	1.05
Oscillatoria	0.02	0.55	0.02	0.00	14.80	16.65	20.97	14.34
Prochlorococcus	0.08	0.00	0.00	0.00	2.00	0.09	0.00	3.73
Leptolyngbya	0.08	0.19	0.00	0.03	14.34	7.24	22.07	7.23
Halomonas	0.00	3.77	5.96	2.94	0.00	0.00	0.10	0.00
Labrenzia	0.13	0.15	0.11	0.00	0.46	2.16	2.09	0.93
Rhodovibrio	0.00	0.00	0.02	0.00	0.23	0.94	0.50	3.15
Chamaesiphon	0.00	0.00	0.00	0.00	0.00	0.19	2.58	1.86
Gloeotrichia	0.00	0.00	0.00	1.07	1.00	2.26	0.99	1.63

Supplementary Table S7 | Assignments of OTUs with at least a two percent relative abundance to genus or next lowest taxonomical unit and their respective relative abundance in each coral sample after six months of transplantation from *S. nigricans*' territories.

**Taxa**	**Coral Sample**								
	1	2	3	4	5	6	7	8	9
Balneatrix	2.16	0.79	2.12	2.12	1.66	4.47	0.14	0.03	1.95
Rhodobacteraceae	2.65	4.86	7.41	8.73	4.62	6.50	0.56	0.39	7.00
Rhodobium	1.94	0.12	1.15	1.92	2.70	1.72	0.14	0.03	2.28
Anderseniella	0.35	2.32	1.02	0.56	0.50	0.23	0.00	0.08	0.39
Pedinomonas	0.00	0.46	0.13	0.70	0.68	0.13	8.11	0.49	1.44
Crenothrix	0.77	2.28	2.81	5.05	3.56	3.70	0.35	0.00	3.00
Prochlorothrix	0.35	0.58	2.12	1.19	0.27	0.08	0.35	0.00	0.30
Pleurocapsa	1.23	0.66	6.16	2.26	2.43	2.93	0.35	0.03	1.23
Nitratireductor	0.09	0.00	0.33	0.31	0.15	0.10	6.01	0.00	0.21
Leptolyngbya	3.81	0.62	5.80	5.69	3.53	5.09	2.52	0.03	5.83
Endozoicomonas	0.07	50.73	0.26	0.07	0.86	0.05	45.98	88.78	0.00
Prochlorococcus	0.04	1.37	1.41	0.45	2.40	0.10	1.12	0.33	0.45
Nannocystineae	3.11	0.75	1.23	1.90	1.96	2.26	0.42	0.05	2.22
Arthrospira	0.57	0.12	2.40	0.22	1.01	0.82	0.70	0.00	0.45
Cystobacterineae	0.62	4.86	1.99	0.47	0.50	0.10	1.40	0.13	0.36
Comamonadaceae	0.90	0.58	0.72	1.10	1.22	2.36	0.84	0.08	5.68
Phyllobacteriaceae	2.78	0.95	0.64	1.22	1.24	0.82	0.07	0.03	0.93
Chroococcidiopsis	0.75	0.29	2.15	2.69	1.72	4.96	0.21	0.00	2.55
Wenxinia	0.82	2.20	1.92	1.19	1.27	0.54	0.77	0.08	0.84
Micrococcus	0.00	0.00	0.00	0.00	0.00	0.00	2.80	0.00	0.00
Oscillatoria	5.31	0.71	1.33	2.89	1.60	11.74	0.63	0.00	0.87
Rhodospirillaceae	8.38	0.62	4.91	6.20	7.47	5.70	0.28	0.15	6.73
Rivularia	4.39	3.57	9.20	11.24	8.45	3.06	0.98	0.05	3.06
Leptospirillum	2.05	0.04	1.28	0.57	1.84	1.49	0.00	0.00	2.16

Supplementary Table S8 | Assignments of OTUs with at least a two percent relative abundance to genus or next lowest taxonomical unit and their respective relative abundance in each coral sample after one year of transplantation from control plots.

**Taxa**	**Coral Sample**								
	1	2	3	4	5	6	7	8	9
Haliea	1.21	0.13	0.00	0.17	0.99	0.19	0.36	0.39	2.03
Balneatrix	4.38	0.28	0.00	0.04	2.13	2.35	1.97	0.42	3.32
Muricauda	1.11	0.04	0.00	0.00	2.57	1.20	0.87	1.00	0.65
Rhodobacteraceae	6.26	0.61	0.00	1.86	7.22	9.39	4.30	3.80	4.50
Pedinomonas	0.25	0.43	0.06	0.72	1.03	3.73	2.02	1.58	1.74
Crenothrix	3.82	0.09	0.00	0.13	0.94	8.49	14.67	1.62	1.10
Pleurocapsa	2.44	0.04	0.00	0.21	1.19	1.61	0.79	0.70	1.27
Ruegeria	1.16	0.17	0.34	0.30	0.92	0.57	0.74	0.93	2.34
Leptolyngbya	9.44	0.19	0.00	0.38	2.59	3.43	2.42	2.06	3.83
Burkholderia	0.03	0.19	0.26	2.53	0.00	0.00	0.00	0.00	0.03
Endozoicomonas	0.06	86.58	94.78	7.34	0.10	0.03	0.02	0.22	0.23
Microcoleus	0.08	0.09	0.00	0.00	0.21	0.03	0.07	0.65	5.40
Geitlerinema	0.83	0.00	0.00	0.00	1.38	1.56	2.07	0.62	0.39
Anabaena	0.76	0.04	0.00	0.04	2.91	0.73	2.06	1.58	0.76
Calothrix	9.47	0.06	0.00	0.00	1.05	0.13	0.63	0.02	0.25
Prochlorococcus	2.29	0.30	0.09	0.51	0.59	1.40	0.86	0.49	0.37
Scherffelia	0.67	0.43	0.40	17.30	0.64	1.47	2.64	2.78	2.22
Nannocystineae	3.30	0.09	0.00	0.08	1.62	0.79	0.70	0.57	3.52
Trichodesmium	0.17	0.00	0.00	0.00	2.36	0.14	0.05	0.00	0.08
Arthrospira	0.33	0.00	0.00	2.95	0.23	0.84	0.10	0.00	0.00
Cystobacterineae	1.07	0.32	2.38	2.49	1.19	0.90	0.51	0.39	0.51
Pseudovibrio	0.00	0.00	0.00	0.00	6.40	0.00	0.21	0.06	0.00
Acinetobacter	0.02	0.04	0.09	34.56	0.00	0.02	0.10	0.12	0.03
Rhodovulum	0.07	0.00	0.00	0.00	0.06	0.06	0.05	3.21	0.11
Comamonadaceae	1.84	0.06	0.00	0.00	0.92	1.28	0.43	0.51	2.05
Chroococcidiopsis	1.88	1.06	0.00	13.59	1.40	3.80	5.95	4.20	1.77
Wenxinia	1.62	0.17	0.00	0.00	1.50	0.98	1.08	3.72	1.21
Pelagibius	0.15	0.09	0.00	0.00	0.39	0.55	0.60	3.74	0.14
Oscillatoria	1.70	0.26	0.00	0.34	1.42	12.15	9.32	19.34	1.32
Rhodospirillaceae	1.63	1.00	0.09	0.08	5.62	4.91	6.61	3.40	3.88
Rivularia	6.97	0.11	0.00	0.34	9.60	10.31	2.40	2.09	5.21

Supplementary Table S9 | Assignments of OTUs with at least a two percent relative abundance to genus or next lowest taxonomical unit and their respective relative abundance in each coral sample after one year of transplantation from *S. apicalis*' territories.

**Taxa**	**Coral Sample**								
	1	2	3	4	5	6	7	8	9
Balneatrix	1.09	0.97	3.33	0.10	0.00	5.66	1.55	0.00	0.38
Muricauda	0.08	0.47	0.00	0.03	0.19	0.80	0.39	2.66	0.28
Rhodobacteraceae	3.71	1.75	3.66	0.00	2.52	9.04	3.79	3.25	3.18
Rhodobium	2.18	0.90	0.29	0.00	0.23	1.02	0.52	0.42	1.62
Crenothrix	1.51	0.00	19.84	0.00	1.21	12.76	1.65	0.67	1.65
Pleurocapsa	1.68	1.98	0.33	0.00	0.14	3.04	2.30	0.25	2.68
Ruegeria	0.72	2.05	0.14	0.23	0.14	1.05	2.78	1.58	1.08
Massilia	0.00	3.92	0.00	0.00	0.05	0.02	0.00	0.58	0.00
Bartonella	0.00	0.00	0.00	0.00	0.00	0.00	0.00	0.00	15.34
Leptolyngbya	1.80	0.50	0.14	0.00	0.14	4.83	3.50	0.58	3.36
Burkholderia	0.00	0.45	3.19	0.30	0.05	0.00	0.13	6.99	0.00
Endozoicomonas	0.47	42.92	58.33	92.70	83.52	0.07	45.21	14.65	0.47
Nitrosococcus	2.56	0.22	0.67	0.03	0.23	0.67	0.16	0.58	1.06
Anabaena	2.00	0.09	0.00	0.00	0.00	0.50	0.19	0.00	5.39
Calothrix	0.06	0.30	1.38	0.00	0.70	5.78	0.13	0.00	0.24
Scherffelia	1.97	0.67	0.00	0.07	0.00	0.20	0.94	15.15	1.06
Cystobacterineae	0.28	0.00	0.00	1.58	0.51	0.12	0.55	2.83	0.59
Acinetobacter	0.04	9.07	0.00	0.03	1.03	0.00	0.00	0.00	0.02
Phyllobacteriaceae	0.72	1.23	0.33	0.00	0.00	0.87	1.23	0.92	2.92
Chroococcidiopsis	3.63	0.24	0.05	0.00	0.00	2.52	3.85	15.40	3.20
Wenxinia	0.09	0.86	0.05	0.00	0.56	2.14	1.88	1.08	0.64
Oscillatoria	16.20	0.00	0.10	0.00	1.68	2.59	0.19	0.58	5.72
Rhodospirillaceae	9.60	1.85	0.00	0.00	0.05	1.54	0.71	0.00	9.22
Ralstonia	0.00	0.13	0.10	0.23	0.00	0.00	0.03	6.16	0.05
Rivularia	0.38	12.81	0.38	0.00	1.40	8.30	1.29	0.17	1.25

Supplementary Table S10 | Assignments of OTUs with at least a two percent relative abundance to genus or next lowest taxonomical unit and their respective relative abundance in each coral sample after one year of transplantation from *S. nigricans*' territories.

**Taxa**	**Coral Sample**									
	1	2	3	4	5	6	7	8	9	10
Rivularia	4.41	0.15	0.30	0.29	0.90	0.27	0.50	0.00	0.85	1.53
Rhodobacteraceae	10.62	12.50	3.62	8.85	7.60	3.43	4.54	0.03	5.41	14.83
Mycoplasma	3.49	0.00	0.00	0.00	0.00	0.00	0.00	0.00	0.00	0.10
Microcoleus	0.49	2.34	0.00	1.89	0.31	0.44	0.79	0.03	0.07	0.10
Chroococcidiopsis	3.10	3.50	1.15	4.89	2.69	2.62	4.68	0.17	3.21	3.92
Anabaena	1.58	0.83	2.93	2.68	0.55	1.62	1.76	0.07	0.68	3.56
Pellia	0.22	0.00	0.00	1.51	0.23	0.10	6.38	0.14	0.00	0.00
Pseudovibrio	0.00	0.00	0.00	0.00	0.00	0.00	0.00	0.06	7.62	0.00
Ruegeria	1.58	0.56	12.20	0.84	2.22	0.34	0.76	0.00	4.31	0.80
Crenothrix	8.55	12.60	0.36	5.13	13.84	3.06	1.08	0.03	1.71	15.23
Rhodobium	0.71	0.95	0.16	1.28	0.31	0.56	1.05	0.00	2.28	0.40
Nannocystineae	5.50	7.20	3.06	4.89	4.33	2.16	2.55	0.03	4.27	5.19
Pleurocapsa	1.74	3.41	0.92	2.42	0.94	0.93	1.87	0.01	2.56	0.93
Calothrix	11.55	2.07	18.45	2.13	1.52	0.07	0.94	0.04	2.85	14.83
Oscillatoria	5.34	19.68	0.13	17.68	1.72	8.06	2.43	0.04	0.71	6.05
Phyllobacteriaceae	2.56	1.31	1.28	0.84	1.72	1.18	1.00	0.07	1.89	0.70
Scherffelia	3.59	0.34	2.43	4.25	4.60	2.28	14.81	0.11	3.03	1.63
Leptolyngbya	1.53	1.36	1.84	2.74	1.01	0.88	1.73	0.01	1.07	1.03
Balneatrix	7.24	3.09	2.93	3.20	2.81	1.59	1.70	0.01	7.51	2.86
Endozoicomonas	0.00	0.51	23.72	0.17	1.48	0.00	0.00	96.25	17.09	0.00
Nitratireductor	0.00	0.00	1.28	0.03	0.04	0.00	0.09	0.00	6.02	0.00
Pedinomonas	1.20	0.02	1.45	2.13	20.47	29.54	3.83	0.00	3.60	2.26
Coleochaete	0.71	0.00	0.07	5.10	13.96	24.74	23.62	0.10	0.00	0.33
Rhodospirillaceae	1.63	4.84	0.69	3.26	1.72	1.27	3.92	0.06	1.89	1.99
Comamonadaceae	1.91	3.21	0.82	3.12	0.70	1.25	0.82	0.01	0.57	1.10

References

S1. Rjaniemi, P. *et al.* Phylogenetic and morphological evaluation of the genera *Anabaena*, *Aphanizomenon*, *Trichormus* and *Nostoc* (Nostocales, Cyanobacteria). *Int. J. Syst. Evol. Microbiol.* **55**, 11-26 (2005).

S2. Blackburn, S. Microalgal applications in bioindustry. *Microbiology Australia* **29**, 30-31 (2008).

S3. Marsac, N. T. *et al.* Photoregulation of gene expression in the filamentous cyanobacterium *Calothrix* sp. PCC 7601: light-harvesting complexes and cell differentiation. *Photosynth. Res*. **18**, 99-132 (1988).

S4. Waterbury, J. & Stanier, R. Two unicellular cyanobacteria which reproduce while budding. *Arch. Microbiol*. **115**, 249-257 (1977).

S5. Fewer, D., Friedl, T. & Budel, B. *Chroococcidiopsis* and heterocyst-differentiating cyanobacteria are each other's closest living relatives. *Mol. Phylogenet. Evol*. **23**, 82-90 (2002).

S6. Delwiche, C. F., Graham, L. E. & Thomson, N. Lignin-like compounds and sporopollenin in *Coleochaete*, an algal model for land plant ancestry. *Science* **245**, 399-401 (1989).

S7. Stoecker, K. *et al.* Cohn's *Crenothrix* is a filamentous methane oxidizer with an unusual methane monooxygenase. *Proc. Natl. Acad. Sci. U.S.A.* **103**, 2363-2367 (2006).

S8. Nakamura, Y. *et al.* Complete genome structure of *Gloeobacter violaceus* PCC 7421, a cyanobacterium that lacks thylakoids. *DNA Res.* **10**, 137-145 (2003).

S9. Goltsman, D. S. A. *et al.* Community genomic and proteomic analyses of chemoautotrophic iron-oxidizing "*Leptospirillum rubarum*" (Group II) and

"*Leptospirillum ferrodiazotrophum*" (Group III) bacteria in acid mine drainage biofilms. *Appl. Environ. Microbiol.* **75**, 4599 (2009).

S10. Karsten, U. & Garcia-Pichel, F. Carotenoids and mycosporine-like amino acids compounds in members of the genus *Microcoleus* (Cyanobacteria): a chemosystematic study. *System. Appl. Microbiol.* **19**, 285-294 (1996).

S11. Klotz, M. G. *et al.* Compete genome sequence of the marine chemolithoautotrophic, ammonia-oxidizing bacterium *Nitrosoccoccus oceani* ATC 19707. *Appl. Environ. Microbiol.* **72**, 6299 (2006).

S12. Turmel, M. *et al.* The complete mitochondrial DNA sequences of *Nephroselmis olivacea* and *Pedinomonas minor*: two radically different evolutionary patterns within green algae. *The Plant Cell* **11**, 1717-1729 (1999).

S13. Odrzykoski, I. J., Chudzinska, E. & Szweykowski, J. The hybrid origin of the polypoid liverwort *Pellia borealis*. *Genetica* **98**, 75-86 (1998).

S14. Wujek, D. E. Intracellular bacteria in the blue-green algae *Pleurocapsa minor*. *T. Am. Microsc. Soc.* **98**, 143-145 (1976).

S15. Rocap, G. *et al.* Genome divergence in two *Prochlorococcus* ecotypes reflects oceanic niche differentiation. *Nature* **424**, 1042-1047 (2003).

S16. Komarek, J. & Komarkova, J. Taxonomic review of the cyanoprokaryotic genera *Planktothrix* and *Planktothricoides*. *Czech Phycology* **4**, 1-18 (2004).

S17. Pinevich, A., Velichko, N. & Ivanikova, N. Cyanobacteria of the genus *Prochlorothrix*. *Front. Microbiol.* **3**, 1-13 (2012).

S18. Acinas, S. G., Haverkamp, T. H. A., Huisman, J. & Stal, L. J. Phenotypic and genetic diversification of *Pseudanabaena* spp. (cyanobacteria). *ISME J.* **3**, 31-46 (2009).

S19. Madigan, M. T. Anoxygenic phototrophic bacteria from extreme environments. *Photosynth. Res.* **76**, 157-171 (2003).

S20. Berrendero, E., Perona, E. & Mateo, P. Genetic and morphological characterization of *Rivularia* and *Calothrix* (Nostocales, Cyanobacteria) from running water. *Int. J. Syst. Evol. Micr.* **58**, 447-460 (2008).

S21. Fuller, N. J. *et al.* Analysis of photosynthetic picoeukaryote diversity at open ocean sites in the Arabian Sea using a PCR biased towards marine algal plastids. *Aquat. Microb. Ecol.* **43**, 79-93 (2006).

S22. Qiang, H., Zarmi, Y. & Richmond, A. Combined effects of light intensity, light-path and culture density on output rate of *Spirulina platensis* (Cyanobacteria). *Eur. J. Phycol.* **33**, 164-171 (1998).

S23. Zouni, A. *et al.* Crystal structure of photosystem II from *Synechococcus elongatus* at 3.8 Å resolution. *Nature* **409**, 739-743 (2000).

S24. Capone, D. G., Zehr, J. P., Paerl, H. W., Bergman, B. & Carpenter, E. J. *Trichodesmium*, a globally significant marine cyanobacterium. *Science* **276**, 1221-1229 (2014).

S25. Urdiain, M. *et al.* Reclassification of *Rhodobium marinum* and *Rhodobium pfennigii* as *Afifella marina* gen. nov. comb. nov. and *Afifella pfennigii* comb. nov., a new genus of photoheterotrophic *Alphaproteobacteria* and emended descriptions of *Rhodobium*, *Rhodobium orientis* and *Rhodobium gokarnense*. *System. Appl. Microbiol.* **31**, 339-351 (2008).

S26. Tsaplina, I. A. *et al.* The dependence of intracellular ATP level on the nutrition mode of the acidophilic bacteria *Sulfobacillus thermotolerans* and *Alicyclobacillus tolerans*. *Microbiol.* **76**, 654-662 (2007).

S27. Brettar, I., Christen, R., Botel, J., Lunsdorf, H. & Hofle, M. G. *Anderseniella baltica* gen. nov., sp. nov., a novel marine bacterium of the *Alphaproteobacteria* isolated from sediment in the central Baltic Sea. *Int. J. Syst. Evol. Micr.* **57**, 2399-2405 (2007).

S28. McIlroy, S. J. *et al.* Metabolic model for the filamentous '*Candidatus* Microthrix parvicella' based on genomic and metagenomic analyses. *ISME J.* **7**, 1161-1172 (2013).

S29. Pfannes, K. R., Vogl, K. & Overmann, J. Heterotrophic symbionts of phototrophic consortia: members of a novel diverse cluster of *Betaproteobacteria* characterized by a tandem *rrn* operon structure. *Environ. Microbiol.* **9**, 2782-2794 (2007).

S30. Spring, S., Lunsdorf, H., Fuchs, B. M. & Tindall, B. J. The photosynthetic apparatus and its regulation in the aerobic gammaproteobacterium *Congregibacter litoralis* gen. nov., sp. nov. *PLoS ONE* **4**, e4866 (2009).

S31. Brinkhoff, T. *et al.* Biogeography and phylogenetic diversity of a cluster of exclusively marine myxobacteria. *ISME J.* **6**, 1260-1272 (2012).

S32. Du, Z., Zhang, W., Xia, H., Lu, G. & Chen, G. Isolation and diversity analysis of heterotrophic bacteria associated with sea anemones. *Acta. Oceanol. Sin*. **229**, 62-69 (2010).

S33. Staley, J. T., Irgens, R. & Brenner, D. J. *Enhydrobacter aerosaccus* gen. nov., sp. nov., a gas-vacuolated, facultatively anaerobic, heterotrophic rod. *Int. J. Syst. Bacter*. **37**, 289-291 (1987).

S34. Angert, E. R & Clements, K. D. Initiation of intracellular offspring in *Epulopiscium*. *Molec. Microbiol*. **51**, 827-835 (2004).

S35. Anderson, R. T. *et al.* Stimulating the in situ activity of *Geobacter* species to remove uranium from the groundwater of a uranium-contaminated aquifer. *Appl. Environ. Microbiol.* **69**, 5884 (2003).

S36. Jeong, S. *et al.* Bacterail community structure in a biofilter used as a pretreatment for seawater desalination. *Ecol. Eng*. **60**, 370-381 (2013).

S37. Jean, W. D., Huang, S.-P., Chen, J.-S. & Shieh, W. Y. *Kangiella taiwanensis* sp. nov. and *Kangiella marina* sp. nov., marine bacteria isolated from shallow coastal water. *Int. J. Syst. Evol. Micr.* **62**, 2229-2234 (2012).

S38. Graca, A. P. *et al.* Antimicrobial activity of heterotrophic bacterial communities form marine sponge *Erylus discophorus* (Astrophorida, Geodiidae). *PLoS ONE* **8**, e78992 (2013).

S39. Kleerebezem, M. *et al.* Complete genome sequence of *Lactobacillus plantarum* WCFS1. *Proc. Natl. Acad. Sci. U.S.A.* **100**, 1990-1995 (2003).

S40. Williams, T. M. & Unz, R. F. The nutrition of *Thiothrix*, type 021N, *Beggiatoa* and *Leucothrix* strains. *Wat. Res.* **23**, 15-22 (1989).

S41. Calugay, R. J. *et al.* Catechol siderophore excretion by magnetotactic bacterium *Magnetospirillum magneticum* AMB-1. *J. Biosci. Bioeng.* **101**, 445-447 (2006).

S42. Amin, S. A., Green, D. H., Al Waheeb, D., Gardes, A. & Carrano, C. J. Iron transport in the genus *Marinobacter*. *Biometals* **25**, 135-147 (2012).

S43. Kampfer, P., Lodders, N., Marin, K. & Falsen, E. Revision of the genus *Massilia* La Scola *et al.* 2000, with an emended description of the genus and inclusion of all species of the genus *Naxibacter* as new combinations, and proposal of *Massilia consociata* sp. nov. *Int. J. Syst. Evol. Micr.* **61**, 1528-1533 (2011).

S44. Kaneko, T. *et al.* Complete genome structure of the nitrogen-fixing symbiotic bacterium *Mesorhizobium loti*. *DNA Res.* **7**, 331-338 (2000).

S45. Patt, T. E., Cole, G. C. & Hanson, R. S. *Methylobacterium*, a new genus of facultatively methylotrophic bacteria. *Int. J. Syst. Bacteriol.* **26**, 226-229 (1976).

S46. Bruns, A., Rohde, M. & Berthe-Corti, L. *Muricauda ruestringensis* gen. nov., sp. nov., a facultatively anaerobic, appendaged bacterium from German North Sea intertidal sediment. *Int. J. Syst. Evol. Micr.* **51**, 1997-2006 (2001).

S47. Hedlund, B. P. & Costa, K. C. [*Neptunomonas*] *Handbook of Hydrocarbon and Lipid Microbiology* [Timmis, K. N. (ed.)] [1773-1779] (Springer-Verlag, Berlin, 2010).

S48. Labbe, N., Parent, S. & Villemur, R. *Nitratireductor aquibiodomus* gen. nov., sp. nov., a novel α-proteobacterium from the marine dentrification system of the Montreal Biodome (Canada). *Int. J. Syst. Evol. Micr.* **54**, 269-273 (2004).

S49. Sakane, T. & Yokota, A. Chemotaxonomic investigation of heterotrophic, aerobic and microaerophilic spirilla, the genera *Aquaspirillum*, *Magnetospirillum* and *Oceanospirillum*. *Syst. Appl. Microbiol.* **17**, 128-134 (1994).

S50. Choi, D. H., Hwang, C. Y. & Cho, B. C. *Pelagibius litoralis* gen. nov., a marine bacterium in the family *Rhodospirillaceae* isolated from coastal seawater. *Int. J. Syst. Evol. Micr.* **59**, 818-823 (2009).

S51. Sun, J., Haveman, S. A., Bui, O., Fahland, T. R. & Lovley, D. R. Constraint-based modeling analysis of the metabolism of two *Pelobacter* species. *BMC Syst. Biol.* **4**, 174 (2010).

S52. Thomas, T. *et al.* Functional genomic signatures of sponge bacteria reveal unique and shared features of symbiosis. *ISME J.* **4**, 1557-1567 (2010).

S53. Cassler, M. *et al.* Use of real-time qPCR to quantify members of the unculturable heterotrophic bacterial community in a deep sea marine sponge, *Vetulina* sp. *Microb. Ecol.* **55**, 384-394 (2008).

S54. Michaud, L. *et al.* Phylogenetic characterization of the heterotrophic bacterial communities inhabiting a marine recirculating aquaculture system. *J. Appl. Microbiol.* **107**, 1935-1946 (2009).

S55. Hiraishi, A., Urata, K. & Satoh, T. A new genus of marine budding phototrophic bacteria, *Rhodobium* gen. nov., which includes *Rhodobium orientis* sp. nov. and *Rhodobium marinum* comb. nov. *Int. J. Syst. Bacter.* **45**, 226-234 (1995).

S56. Martens, T. *et al.* Reclassification of *Roseobacter gallaeciensis* Ruiz-Ponte *et al.* 1998 as *Phaeobacter gallaeciensis* gen. nov., comb. nov., description of *Phaeobacter inhibens* sp. nov., reclassification of *Ruegeria algicola* (Lafay *et al.* 1995) Uchino *et al.* 1999 as *Marinovum algicola* gen. nov., comb. nov., and emended descriptions of the genera *Roseobacter*, *Ruegeria* and *Leisingera*. *Int. J. Syst. Evol. Microbiol.* **56**, 1293-1304 (2006).

S57. Madigan, M., Cox, S. S. & Stegeman, R. A. Nitrogen fixation and nitrogenase activities in members of the family *Rhodospirillaceae*. *J. Bacteriol.* **157**, 73 (1984).

S58. Khatri, I., Nupur Korpole, S., Subramanian, S. & Pinnaka, A. K. Draft genome sequence of *Rhodovulum* sp. strain PH10, a phototrophic Alphaproteobacterium isolated from a soil sample of mangrove of Namkhana, India. *J. Bacteriol.* **194**, 6363 (2012).

S59. Mosso, M. A., de la Rosa, M. C., Vivar, C. & Medina, M. R. Heterotrophic bacterial populations in the mineral waters of thermal springs in Spain. *J. Appl. Bacteriol.* **77**, 370-381 (1994).

S60. Glockner, F. O. *et al.* Complete genome sequence of the marine planctomycete *Pirellula* sp. strain 1. *Proc. Nat. Acad. Sci. U.S.A.* **100**, 8298-8303 (2003).

S61. Hyun, D.-W. *et al.* Genome sequence of the moderately halophilic bacterium *Salinicoccus carnicancri* type strain Crm^T (=DSM 23852^T). Stand. *Genomic Sci.* **8**, 255-263 (2013).

S62. Lopez-Lopez, A. *et al.* *Thalassospira lucentensis* gen. nov., sp. nov., a new marine member of the *α-Proteobacteria. Int. J. Syst. Evol. Micr.* **52**, 1277-1283 (2002).

S63. Ying, J.-Y. *et al.* *Wenxinia marina* gen. nov., sp. nov., a novel member of the *Roseobacter* clade isolated from oilfield sediments of the South China Sea. *Int. J. Syst. Evol. Micr.* **57**, 1711-1716 (2007).

S64. Werren, J. H. Biology of *Wolbachia*. *Annu. Rev. Entomol*. **42**, 587-609 (1997).

S65. Bergogne-Berezin, E. & Towner, K. J. *Acinetobacter* spp. as nosocomial pathogens: microbiological, clinical, and epidemiological features. *Clin. Microbiol. Rev*. **9**, 148-165 (1996).

S66. Janda, J. M. & Abbott, S. L. Evolving concepts regarding the genus *Aeromonas*: an expanding panorama of species, disease presentations, and unanswered questions. *Clin. Infect. Dis*. **27**, 332-344 (1998).

S67. Dauga, C. *et al. Balneatrix alpica* gen. nov., sp. nov., a bacterium associated with pneumonia and meningitis in a spa therapy centre. *Res. Microbiol*. **144**, 35-46 (1993).

S68. Anderson, B. E. & Neuman, M. A. *Bartonella* spp. As emerging human pathogens. *Clin. Microbiol. Rev.* **10**, 203-219 (1997).

S69. Govan, J. R. & Deretic, V. Microbial pathogenesis in cystic fibrosis: mucoid *Pseudomonas aeruginosa* and *Burkholderia cepacia*. *Microbiol. Rev*. **60**, 539 (1996).

S70. Schmitz-Esser, S. *et al.* The genome of the amoeba symbiont "*Candidatus* Amoebophilus asiaticus" reveals common mechanisms for host cell interaction among amoeba-associated bacteria. *J. Bacteriol*. **192**, 1045 (2010).

S71. Calderon, G. *et al. Chryseobacterium indologenes* infection in a newborn: a case report. *J. Med. Case Rep*. **5**, 10 (2011).

S72. Freeman, V. J. Studies of the virulence of bacteriophage-infected strains of *Corynebacterium diphtheriae. J. Bacteriol*. **61**, 675-688 (1951).

S73. Xu, H.-S. *et al.* Survival and viability of nonculturable *Escherichia coli* and *Vibrio cholerae* in the estuarine and marine environment. *Microb. Ecol*. **8**, 313-323 (1982).

S74. Myers, J. L., Sekar, R. & Richardson, L. L. Molecular detection and ecological significance of the cyanobacterial genera *Geitlerinema* and *Leptolyngbya* in black band disease of corals. *Appl. Environ. Microbiol.* **73**, 5173-5182 (2007).

S75. Arenskotter, M., Broker, D. & Steinbuchel, A. Biology of the metabolically diverse genus *Gordonia*. *Appl. Environ. Microbiol.* **70**, 3195 (2004).

S76. Stevens, D. A., Hamilton, J. R., Johnson, N., Kim, K. K. & Lee, J.-S. *Halomonas*, a newly recognized human pathogen causing infections and contamination in a dialysis center: Three new species. *Medicine* **88**, 244-249 (2009).

S77. Kloos, W. E. & Musselwhite, M. S. Distribution and persistence of *Staphylococcus* and *Micrococcus* species and other aerobic bacteria on human skin. *Appl. Environ. Microbiol.* **30**, 381 (1975).

S78. Tully, J. G., Taylor-Robinson, D., Cole, R. M. & Rose, D. L. A newly discovered mycoplasma in the human urogenital tract. *Lancet.* **1**, 1288-1291 (1981).

S79. Brook, I. & Frazier, E. H. Infections caused by *Propionibacterium* species. *Clin. Infect. Dis*. **13**, 815-818 (1991).

S80. Stover, C. K. *et al.* Complete genome sequence of *Pseudonomas aeruginosa* PA01, an opportunistic pathogen. *Nature* **406**, 959-964 (2000).

S81. Salanoubat, M. *et al.* Genome sequence of the plant pathogen *Ralstonia solanacearum*. *Nature* **415**, 497-502 (2002).

S82. Richardson, L. L. *et al.* Florida's mystery coral-killer identified. *Nature* **392**, 557-558 (1998).

S83. Lowy, F. D. *Staphylococcus aureus* infections. *New Engl. J. Med*. **339**, 520-532 (1998).
